# Tabetri™ (*Tabebuia avellanedae* Ethanol Extract) Ameliorates Osteoarthritis Symptoms Induced by Monoiodoacetate through Its Anti-Inflammatory and Chondroprotective Activities

**DOI:** 10.1155/2017/3619879

**Published:** 2017-11-26

**Authors:** Jae Gwang Park, Young-Su Yi, Yo Han Hong, Sulgi Yoo, Sang Yun Han, Eunji Kim, Seong-Gu Jeong, Adithan Aravinthan, Kwang Soo Baik, Su Young Choi, Young-Jin Son, Jong-Hoon Kim, Jae Youl Cho

**Affiliations:** ^1^Department of Genetic Engineering, Sungkyunkwan University, Suwon 16419, Republic of Korea; ^2^Department of Pharmaceutical Engineering, Cheongju University, Cheongju 28503, Republic of Korea; ^3^College of Veterinary Medicine, Chonbuk National University, Iksan 54596, Republic of Korea; ^4^R&I Planning Department, Nutribiotech Co. Ltd., Seoul 06132, Republic of Korea; ^5^Department of Pharmacy, Sunchon National University, Suncheon 57922, Republic of Korea

## Abstract

Although osteoarthritis (OA), a degenerative joint disease characterized by the degradation of joint articular cartilage and subchondral bones, is generally regarded as a degenerative rather than inflammatory disease, recent studies have indicated the involvement of inflammation in OA pathogenesis. *Tabebuia avellanedae* has long been used to treat various diseases; however, its role in inflammatory response and the underlying molecular mechanisms remain poorly understood. In this study, the pharmacological effects of Tabetri (*Tabebuia avellanedae* ethanol extract (Ta-EE)) on OA pathogenesis induced by monoiodoacetate (MIA) and the underlying mechanisms were investigated using experiments with a rat model and *in vitro* cellular models. In the animal model, Ta-EE significantly ameliorated OA symptoms and reduced the serum levels of inflammatory mediators and proinflammatory cytokines without any toxicity. The anti-inflammatory activity of Ta-EE was further confirmed in a macrophage-like cell line (RAW264.7). Ta-EE dramatically suppressed the production and mRNA expressions of inflammatory mediators and proinflammatory cytokines in lipopolysaccharide-stimulated RAW264.7 cells without any cytotoxicity. Finally, the chondroprotective effect of Ta-EE was examined in a chondrosarcoma cell line (SW1353). Ta-EE markedly suppressed the mRNA expression of matrix metalloproteinase genes. The anti-inflammatory and chondroprotective activities of Ta-EE were attributed to the targeting of the nuclear factor-kappa B (NF-*κ*B) and activator protein-1 (AP-1) signaling pathways in macrophages and chondrocytes.

## 1. Introduction

Osteoarthritis (OA) is a time- and age-dependent progressive degenerative joint disease characterized by the degradation of joint cartilage and the underlying bones. OA is characterized by joint stiffness and pain caused by articular cartilage damage, the alteration of subchondral bones, formation of osteophytes, and thickening of synovial linings [1–4]. One of the major risk factors for OA is age. Most OA patients are over 45 years of age, and the highest morbidity attributed to OA is observed in patients over 60 years of age [5]. Among adults over 60 years of age, the prevalence of OA is approximately 10% in males and 13% in females [6]. Given the current worldwide demographic trend in which the older population is growing quickly, OA patients are expected to increase in the future. Despite this large number of OA patients, no disease-modifying drugs have been developed to effectively treat OA, and the available drugs only alleviate OA symptoms [7]. Therefore, joint replacement is the only treatment available to OA patients who reach the final stage of OA, highlighting the urgent need to develop effective anti-OA drugs. One of the unresolved issues in OA pathogenesis is the contribution of inflammatory response and oxidative stress to the onset and progression of OA. These two risk factors have been increasingly considered to be closely integrated during the pathogenesis of OA. A number of inflammatory mediators, including proinflammatory cytokines, chemokines, growth factors, and prostaglandin E_2_ (PGE_2_), have been reported to be increased in the joint tissues of OA patients and OA-like animals [8–10]. Inflammatory responses along with other risk factors, such as aging and mechanical load, were reported to induce oxidative stress by production of nitric oxide (NO), reactive oxygen species (ROS), hydrogen peroxide (H_2_O_2_), superoxide anion, and peroxynitrite and also to suppress the expression of the enzymes responsible for antioxidant and ROS scavenging activities [11]. Moreover, oxidative stress has been reported to cause abnormality in the metabolisms of cartilages and bones, exacerbating the degradation and overall reparative potential of osteoblasts, chondrocytes, and their precursor cells [11, 12]. Although OA has long been regarded as a degenerative rather than inflammatory joint disease, recent studies have reported correlations between OA pathogenesis and inflammatory responses as well as oxidative stress [10, 13–20].

Inflammation, which is a series of complex biological processes to prevent the body from various invading pathogens and environmental dangers, is characterized by redness, heat, swelling, pain, and loss of function [21–24]. Although an inflammatory response is a defense mechanism, chronic inflammation resulting from repeated and prolonged inflammatory response is thought to be a major risk factor for various inflammatory/autoimmune diseases [25, 26]. An inflammatory response is initiated when pattern recognition receptors recognize pathogen-associated molecular patterns expressed on the inflammatory cells via the activation of inflammatory signaling pathways, including nuclear factor-kappa B (NF-*κ*B), activated protein-1 (AP-1), and interferon-regulatory factors. This leads to the production of proinflammatory cytokines and genes such as tumor necrosis factor-alpha, interleukin (IL)-1*β*, IL-6, inducible nitric oxide synthase (iNOS), and cyclooxygenase-2 (COX-2) along with inflammatory mediators such as PGE_2_ and nitrites [27–30].

*Tabebuia avellanedae* Lorentz ex Griseb (Bignoniaceae) is a tree that belongs to the plant family Bignoniaceae of the genus *Tabebuia*. It is found in the tropical rain forests of South America, particularly in Brazil. The product produced from the inner, purple-colored bark of this tree is known as Taheebo or pau d'arco and has been used pharmacologically to treat various conditions including fungal infection, eczema, psoriasis, and skin cancer [31–33]. Various active pharmacological compounds such as naphthoquinones, furanonaphthoquinones, anthraquinones, *β*-lapachone, benzoic acid derivatives, benzaldehyde derivatives, cyclopentene derivatives, iridoids, coumarins, anthraquinone-2-carboxlic acid, and flavonoids have been extracted from *Tabebuia avellanedae* [34–43]. Recent studies found that some of these compounds exert anti-inflammatory, antibacterial, and anticancer activity [36, 37, 41, 44–46]. However, few studies have explored the effects of *Tabebuia avellanedae* on inflammatory response and inflammatory diseases.

In this study, we prepared the ethanol extract of *Tabebuia avellanedae* (Ta-EE, Tabetri) and investigated (1) its *in vivo* pharmacological effects on the pathogenesis of OA using monoiodoacetate- (MIA-) induced OA rats as an experimental animal model and (2) its underlying molecular mechanisms using two *in vitro* models: a lipopolysaccharide- (LPS-) challenged macrophage-like cell line (RAW264.7) and a phorbol 12-myristate 13-acetate- (PMA-) challenged chondrosarcoma cell line (SW1353).

## 2. Materials and Methods

### 2.1. Materials

Ta-EE, *Perna canaliculus* (green lipped mussel) lipid extract (Pc-LE), and methylsulfonylmethane (MSM) were kindly provided from Nutribiotech Co. Ltd. (Seoul, South Korea). LPS (*E. coli* 0111:B4), PMA, 3-(4,5-dimethylthiazol-2-yl)-2,5-diphenyltetrazolium bromide (MTT), sodium dodecyl sulfate (SDS), monoiodoacetate (MIA), safranin O, toluidine blue, hematoxylin, and eosin were purchased from Sigma Chemical Co. Ltd. (St. Louis, MO, USA). RAW264.7 and SW1353 cells were purchased from American Type Culture Collection (Rockville, MD, USA). Roswell Park Memorial Institute (RPMI) 1640, *Dulbecco's modified Eagle's medium* (*DMEM*), fetal bovine serum (FBS), phosphate buffered saline (PBS), streptomycin, penicillin, and L-glutamine were purchased from Gibco (Grand Island, NY, USA). Enzyme-linked immunosorbent assay (ELISA) kits for PGE_2_, LTB_4_, IL-1*β*, and IL-6 were purchased from R&D Systems (Minneapolis, MN, USA). TRI Reagent® was purchased from Molecular Research Center Inc. (Cincinnati, OH, USA). MuLV reverse transcriptase was purchased from Thermo Fisher Scientific (Waltham, MA, USA). The primers used for quantitative real-time polymerase chain reaction (PCR) and semiquantitative PCR were synthesized, and the PCR premix for semiquantitative PCR was purchased from Bioneer Inc. (Daejeon, South Korea). pPCRBIO SyGreen Mix for quantitative real-time PCR was purchased from PCR Biosystems Ltd. (London, United Kingdom). Antibodies specific for the phosphorylated and total forms of p85, IKK*α*/*β*, I*κ*Bα, p50, p65, p38, JNK, ERK, c-Jun, c-Fos, Src, Syk, IRAK4, and *β*-actin were purchased from Cell Signaling Technology (Beverly, MA, USA). An enhanced chemiluminescence system was purchased from AbFrontier (Seoul, South Korea).

### 2.2. Cell Culture

The macrophage-like cell line (RAW264.7) and chondrosarcoma cell line (SW1353) were cultured in RPMI 1640 media and DMEM, respectively, containing 10% heat-inactivated FBS, 100 mg/ml streptomycin, 100 U/ml penicillin, and 2 mM L-glutamine at 37°C in a 5% CO_2_-humidified incubator. Two times per week, the culture media were replaced, and the cells were subcultured.

### 2.3. Animals and Husbandry

Sprague-Dawley rats (male, five weeks old) were purchased from Daehan Biolink (Osong, South Korea). The rats (three rats/cage) were maintained in the plastic cages at room temperature with constant 12 h : 12 h light and dark cycles, and the rats were fed tap water and pelleted foods (Samyang, Daejeon, South Korea) ad libitum. All studies using these rats were conducted according to the guidelines of the Institutional Animal Care and Use Committee at Sungkyunkwan University.

### 2.4. Induction and Monitoring of MIA-Induced OA in Rats

**Figure 1:**
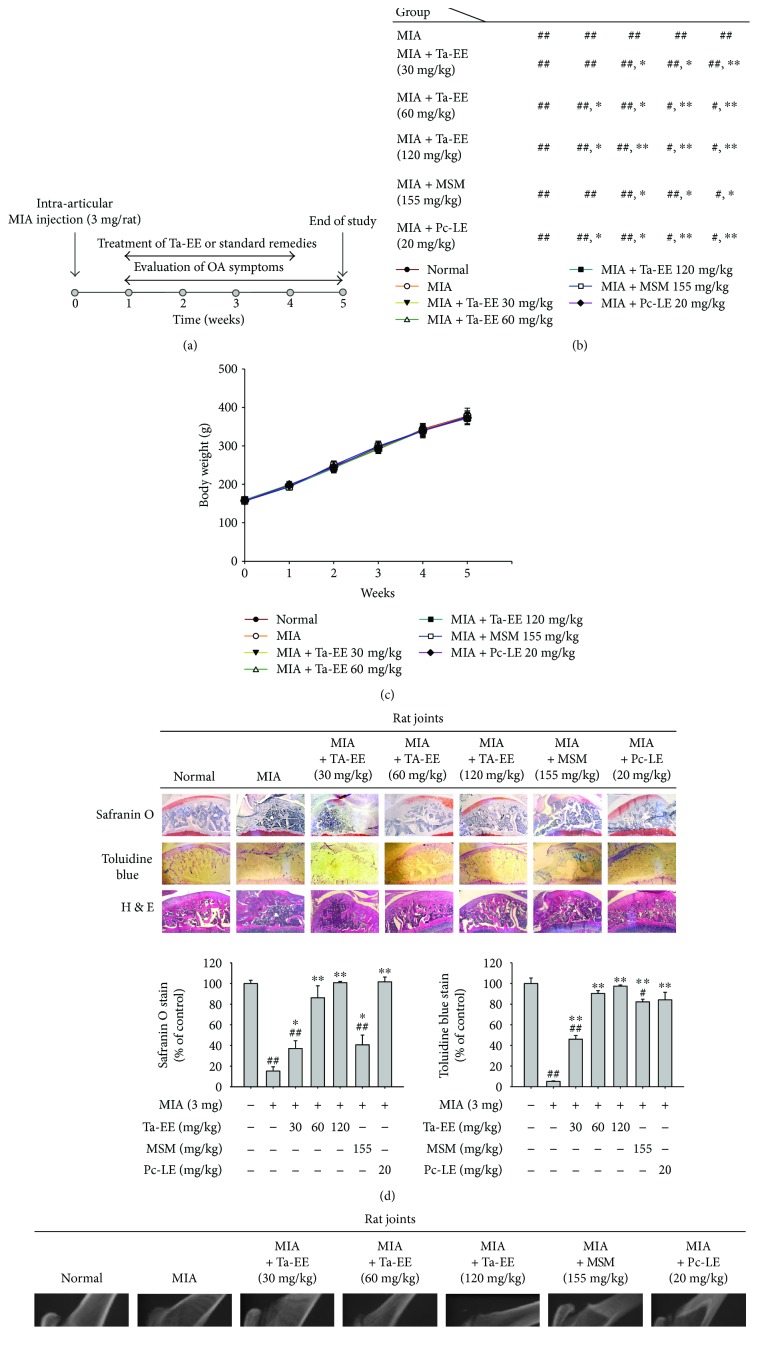
Ta-EE ameliorated OA symptoms in MIA-induced OA rats. (a) Experimental schedule of OA induction, treatment, and evaluation. Sprague-Dawley rats (10 rats/group) were intra-articularly injected with MIA (3 mg/rat). One week later, the rats were subjected to different treatments once a day for four weeks. The study finished at week five. (b) MIA-induced OA rats were orally administered with either Ta-EE (30–120 mg/kg), MSM (155 mg/kg), or Pc-LE (20 mg/kg) once a day for four weeks, and paw withdrawal threshold was measured once a week for five weeks. The significance of each group with respect to the MIA group each week is summarized in the box. (c) Body weights of the rats were measured once a week for five weeks. ((d); left panel) MIA-induced OA rats were orally administered with Ta-EE (30–120 mg/kg), MSM (155 mg/kg), or Pc-LE (20 mg/kg) once a day for four weeks, and the tibia bones were stained with hematoxylin and eosin, safranin O, or toluidine blue at week five. The areas stained by ((d); middle panel) safranin O and ((d); right panel) toluidine blue were measured and plotted. (e) The femur and tibia bones of the MIA-induced OA rats administered with Ta-EE (30–120 mg/kg), MSM (155 mg/kg), or Pc-LE (20 mg/kg) were analyzed by X-ray radiography. ^∗^*P* < 0.05 and ^∗∗^*P* < 0.005 versus a control group; ^#^*P* < 0.05 and ^##^*P* < 0.005 versus a normal group.

MIA-induced OA was induced in the Sprague-Dawley rats as described previously [47]. The rats were anesthetized using an exposure chamber with ether. The hair of the anesthetized rats was shaved and the skins were sanitized with 70% ethanol. The rats were injected with 3 mg of MIA in 50 *μ*l sterile PBS into the knee joint cavity (intra-articularly). The needle was passed through the patellar tendons of the rats, and rats were then transferred to cages. After one week, the MIA-injected rats were randomly assigned to different treatment groups (three rats/cage) and orally administered with either three different doses of Ta-EE (30, 60, and 120 mg/kg), MSM (155 mg/kg), or Pc-LE (20 mg/kg) in 100 *μ*l once a day for four weeks. The pharmacological effects of Ta-EE on OA symptoms were evaluated once a week for five weeks, and the study was ended at week five (Figure 1(a)).

### 2.5. Measurement of Paw Withdrawal Threshold

Pain sensitivity was evaluated by measuring the withdrawal threshold of the paw in the direction that caused osteoarthritis in response to mechanical stimuli with von Frey hairs. Before evaluation, the animals were placed in a plastic cage with a wire-net floor and allowed to acclimate for 10 min. A series of eight calibrated von Frey hairs (2, 4, 6, 8, 10, 15, 26, and 60 g) were applied to the plantar surface of the hind paw. Each von Frey hair was held for 2 s with 3 min between measurements. The pain sensitivity was inversely proportional to the response to the intensity of von Frey hair.

### 2.6. Measurement of Body Weight

The body weights of all rats in each experimental group were measured once a week for five weeks using a scale (Mettler Toledo, Columbus, OH, USA).

### 2.7. Hematoxylin and Eosin and Immunohistochemical Staining

The cartilage sample was fixed with 10% buffered formaldehyde solution and then embedded in paraffin. Sections of cartilage (thickness = 4 *μ*m) were stained with hematoxylin-eosin to observe pathological changes, safranin O to observe cartilage, or toluidine blue to observe glycosaminoglycans (GAGs), as reported previously [48]. The animals were scanned by microcomputed tomography (Chonbuk University, Icksan, South Korea) to observe architectural changes in the femur and tibia bones four weeks after intraarticular injection.

### 2.8. X-Ray Radiography

The degradation of cartilage and subchondral bones was evaluated by X-ray radiography in one representative rat in each experimental group.

### 2.9. PGE_2_ Production Assay

RAW264.7 cells (1 × 10^6^ cells/ml) pretreated with Ta-EE (0–300 *μ*g/ml) for 30 min were treated with LPS (1 *μ*g/ml) for 24 h. Rats intra-articularly injected with MIA (3 mg) were orally administered with either Ta-EE, MSM, or Pc-LE for four weeks. The production and release of PGE_2_ in the cell culture medium and rat sera were determined by enzyme immunoassay as described previously [49].

### 2.10. ELISA

The secreted levels of LTB_4_, IL-1*β*, and IL-6 in (1) the sera of rats intra-articularly injected with MIA (3 mg) and orally administered with either Ta-EE, MSM, or Pc-LE for four weeks and (2) the cell culture media of RAW264.7 cells pretreated with Ta-EE (0–300 *μ*g/ml) for 30 min and treated with LPS (1 *μ*g/ml) for 24 h were determined by ELISA according to the manufacturer's instruction.

### 2.11. Nitrite Production Assay

RAW264.7 cells (5 × 10^6^ cells/ml) pretreated with either Ta-EE (0–300 *μ*g/ml) for 30 min were treated with LPS (1 *μ*g/ml) for 24 h. Nitrite production was determined by measuring the nitrite level in the cell culture medium using Griess reagents as previously described [26].

### 2.12. Cell Viability Assay

The cytotoxic effects of Ta-EE on RAW264.7 cells (1 × 10^6^ cells/ml) and SW1353 cells (1 × 10^6^ cells/ml) were determined by treating the cells with escalating doses of Ta-EE (0, 75, 100, and 300 *μ*g/ml) for 24 h, and cell viability was determined by MTT assay as reported previously [50, 51]. Briefly, the cell culture media were mixed with MTT solution (10 mg/ml in PBS, pH 7.4) and incubated at 37°C for 4 h. A 15% SDS solution was directly added to the mixture to stop the reaction followed by incubation at 37°C for 24 h. Optical density was then measured at 570 nm using a Spectramax 250 microplate reader.

### 2.13. Quantitative and Semiquantitative PCR

To determine the mRNA expression levels of IL-1*β*, iNOS, and COX-2, total RNA was extracted from RAW264.7 cells treated with Ta-EE (0–300 *μ*g/ml) and LPS (1 *μ*g/ml) for 6 h using TRI reagent® according to the manufacturer's instructions and immediately stored at −70°C until use. Total cDNA was synthesized from 1 *μ*g of total RNA using MuLV reverse transcriptase according to the manufacturer's instructions, and quantitative real-time and semiquantitative PCR were conducted as described previously [26]. The nucleic acid sequences of the primers used for PCR are listed in Table 1.

### 2.14. Western Blot Analysis

For preparing total cell lysates, RAW264.7 and SW1353 cells (1 × 10^6^ cells/ml) were lysed with ice-cold lysis buffer (20 mM Tris-HCl, pH 7.4, 2 mM EDTA, 2 mM EGTA, 50 mM glycerol phosphate, 1 mM DTT, 2 *μ*g/ml aprotinin, 2 *μ*g/ml leupeptin, 1 *μ*g/ml pepatatin, 50 *μ*M PMSF, 1 mM benzamide, 2% Triton X-100, 10% glycerol, 0.1 mM sodium vanadate, 1.6 mM pervanadate, and 20 mM NaF), and the total cell lysates were clarified by centrifugation at 12,000 rpm for 5 min at 4°C and stored at −20°C until use.

For Western blot analysis, the total cell lysates were electrophoresed on SDS-polyacrylamide gels, and the total proteins in the gel were transferred to a polyvinylidene difluoride membrane by electroblotting. The protein-transferred membrane was blocked using 5% BSA in PBS at room temperature for 1 h followed by incubation with the primary antibodies specific for each target for 1 h at room temperature. After incubation with primary antibodies, the membranes were washed three times (10 min each time) with 0.1% TBST (Tris-base, NaCl, 0.1% Tween 20, pH 7.6), incubated with HRP-linked secondary antibodies containing 3% BSA for 1 h at room temperature, and washed with 0.1% TBST (three times for 10 min each). The phosphorylated and total forms of p85, IKK*α*/*β*, I*κ*B*α*, p50, p65, p38, JNK, ERK, c-Jun, c-Fos, Src, Syk, IRAK4, and *β*-actin were visualized using an enhanced chemiluminescence (ECL) reagent according to the manufacturer's instructions.

### 2.15. *In Vitro* Kinase Assay

The *in vitro* effects of Ta-EE on the activities of purified target kinases (Syk, Src, and IRAK4) were determined using the kinase profiler service of Millipore (Billerica, MA, USA) as reported previously [52]. Briefly, each kinase was incubated with reaction buffer containing MgATP for 40 min at room temperature and further incubated after adding 3% phosphoric acid solution to the reaction solution. Each incubate was dropped onto a P30 filtermat and washed three times with phosphoric acid (5 min each wash) and once with methanol for 5 min. After drying the incubates, the kinase activities were measured by scintillation counting.

### 2.16. Statistical Analysis

All data are expressed as the mean and standard deviation of at least three independent experiments. For statistical significance, results were analyzed by analysis of variance/Scheffe's post hoc test and Kruskal-Wallis/Mann–Whitney *U* test, and *P* < 0.05 was considered statistically significant. All statistical analyses were conducted using SPSS.

## 3. Results and Discussion

Despite several studies reporting the anti-inflammatory activity of *Tabebuia avellanedae* [41–44, 46, 53, 54], its pharmacological effects on inflammatory diseases along with their underlying molecular mechanisms remain poorly understood. To address this gap in knowledge, we investigated the pharmacological effects of Ta-EE on the pathogenesis of OA using MIA rats as an animal model and examined the molecular mechanism of Ta-EE-mediated anti-inflammatory activity using *in vitro* cell line models.

The experimental design used to explore the *in vivo* pharmacological effects of Ta-EE on OA pathogenesis using experimental OA rats is shown in Figure 1(a) and detailed in Sections 2.4 and 2.5. The mechanical paw withdrawal threshold to von Frey stimuli as a pain indicator was significantly increased in the OA rats administered with Ta-EE compared to the untreated OA rats (Figure 1(b)). Ta-EE exerted a comparable effect with MSM and Pc-LE (positive controls; Figure 1(b)), suggesting that Ta-EE could compete with the anti-inflammatory therapeutic compounds currently used as anti-inflammatory agents. Interestingly, the effect of Ta-EE on paw withdrawal threshold was not dose dependent, indicating that the pharmacological benefit of Ta-EE can be achieved with small doses, which could decrease toxicity and production cost. The rats in all groups tolerated the treatments well and did not show any decrease in body weight during treatment with Ta-EE and the two positive controls (Figure 1(c)), indicating that Ta-EE does not exhibit *in vivo* toxicity or cause side effects at the doses studied herein.

The pharmacological effect of Ta-EE on OA pathogenesis was investigated by histopathological analyses. The degradation of articular cartilage was dramatically inhibited in the rats administered with Ta-EE and the two positive controls, while the articular cartilage of OA rats was severely degraded (Figure 1(d); left panel). Interestingly, the chondroprotective effect by Ta-EE was better than MSM at the doses of 60 and 120 mg/kg (Figure 1(d); middle and right panels). Hematoxylin and eosin staining supported these observations; the damage to cartilage and subchondral bone was markedly reduced in the OA rats administered with Ta-EE and the positive controls, whereas tissue damage was severe in untreated OA rats (Figure 1(d); left panel). The cartilage areas stained by safranin O (Figure 1(d); middle panel) and toluidine blue (Figure 1(d); right panel) were measured and plotted. Unlike the paw withdrawal threshold results, Ta-EE inhibited cartilage degradation in a dose-dependent manner. The reason for this difference is unclear and requires further investigation; however, it might be attributed to the different pathophysiological mechanisms that induce paw pain and articular cartilage degradation. The degradation of articular cartilage and subchondral bones was further evaluated in the OA rats administered with Ta-EE by an X-ray radiography, and the results confirmed the findings of the histopathological analyses; Ta-EE and the two positive controls effectively inhibited the degradation of articular cartilage and subchondral bones, while these tissues were severely degraded in untreated OA rats (Figure 1(e)). In accordance with the histopathological analyses (Figure 1(d)), Ta-EE showed better protective effect on the degradation of articular cartilage and subchondral bones at the doses of 60 and 120 mg/kg than MSM (Figure 1(e)). These *in vivo* results strongly suggest that Ta-EE significantly ameliorates the symptoms of OA and exhibits effective chondro- and osteoprotective activity in OA pathogenesis.

**Figure 2:**
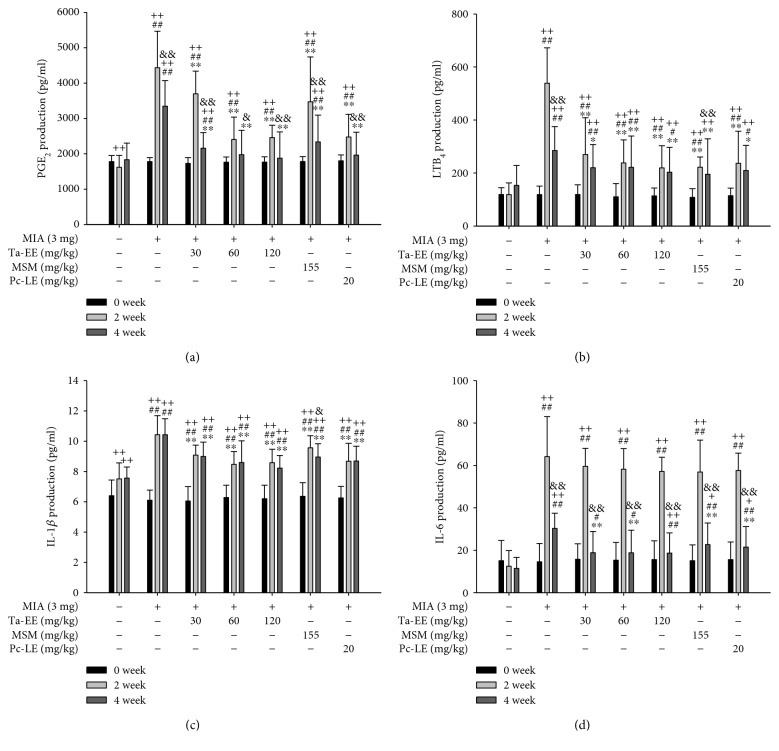
Ta-EE reduced the serum levels of inflammatory mediators and proinflammatory cytokines in MIA-induced OA rats. MIA-induced OA rats were orally administered with either Ta-EE (30–120 mg/kg), MSM (155 mg/kg), or Pc-LE (20 mg/kg) once a day, and the levels of (a) PGE_2_, (b) LTB_4_, (c) IL-1*β*, and (d) IL-6 in the sera of the rats in each group were measured at weeks zero, two, and four. ^∗^*P* < 0.05, ^∗∗^*P* < 0.005 versus a control group; ^#^*P* < 0.05 and ^##^*P* < 0.005 versus a normal group; ^+^*P* < 0.05 and ^++^*P* < 0.005 week 0 versus week 2 or 4; ^&^*P* < 0.05 and ^&&^*P* < 0.005 week 2 versus week 4.

As mentioned earlier, several recent studies have reported a functional correlation between inflammation and OA pathogenesis [8–16]. Therefore, we examined the *in vivo* effects of Ta-EE on the production of inflammatory mediators and proinflammatory cytokines in the sera of OA rats. The serum levels of PGE_2_ (Figure 2(a)), LTB_4_ (Figure 2(b)), and IL-1*β* (Figure 2(c)) were significantly lower in the OA rats administered with Ta-EE compared to untreated OA rats at both two and four weeks (*P* < 0.005) after administration. In contrast, the serum level of IL-6 was significantly lower in the OA rats administered with Ta-EE compared to untreated OA rats at only four weeks after administration (*P* < 0.05, Figure 2(d)). Although IL-1*β* and IL-6 are both proinflammatory cytokines, the effects of Ta-EE on the IL-1*β* and IL-6 levels in the sera of OA rats differed, and the reason for this difference is unclear. However, similar patterns were observed in the sera of mice in which an inflammatory gene had been deleted (Yi et al., unpublished data), demonstrating that the production and metabolism of different proinflammatory cytokines can be differentially regulated by anti-inflammatory agents. Anti-inflammatory effect of Ta-EE on the production of these molecules was further compared with two positive controls. Ta-EE exerted better suppressive effect than MSM for the production of PGE_2_ and IL-1*β* on week two at both 60 and 120 mg/kg doses and on week four at a 120 mg/kg dose (Figures 2(a) and 2(c)), whereas the effect of Ta-EE was similar with that of MSM for the production of LTB_4_ and IL-6 (Figures 2(b) and 2(d)). In contrast, its suppressive effect on the production of these molecules was comparable to that of Pc-LE on all weeks at both doses (Figures 2(a), 2(b), 2(c), and 2(d)). These results suggest that Ta-EE has a better anti-inflammatory activity than MSM which is currently using as an anti-inflammatory agent and that Ta-EE might be a promising anti-inflammatory remedy for various inflammatory diseases.

**Figure 3:**
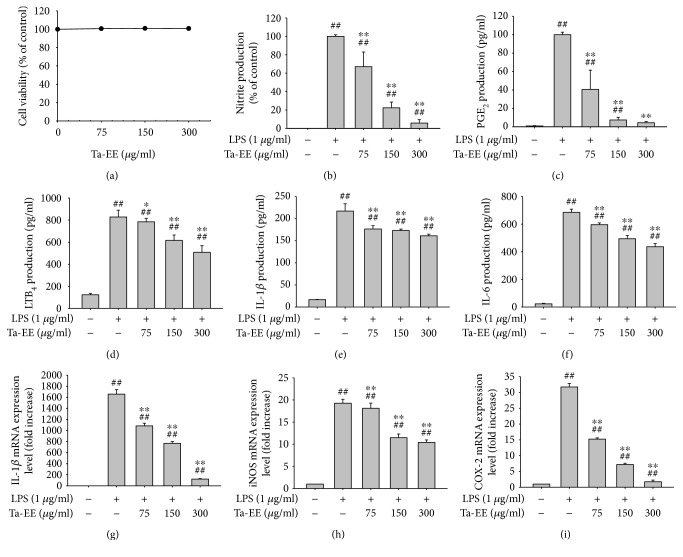
Ta-EE suppressed the production and mRNA expressions of inflammatory mediators and proinflammatory cytokines in LPS-stimulated RAW264.7 cells. (a) RAW264.7 cells were treated with Ta-EE (0–300 *μ*g/ml) for 24 h, and cell viability was measured by MTT assay. (b) RAW264.7 cells pretreated with Ta-EE (0–300 *μ*g/ml) for 30 min were treated with LPS (1 *μ*g/ml) for 24 h, and nitrite production levels in the cell culture media were measured using Griess reagents. RAW264.7 cells pretreated with Ta-EE (0–300 *μ*g/ml) for 30 min were treated with LPS (1 *μ*g/ml) for 24 h, and the production levels of (c) PGE_2_, (d) LTB_4_, (e) IL-1*β*, and (f) IL-6 in the cell culture media were measured by ELISA. RAW264.7 cells pretreated with Ta-EE (0–300 *μ*g/ml) for 30 min were treated with LPS (1 *μ*g/ml) for 6 h, and the mRNA expression levels of (g) IL-1*β*, (h) IL-6, and (i) COX-2 in the cells were measured by quantitative real-time PCR. ^∗^*P* < 0.05 and ^∗∗^*P* < 0.005 versus a control group; ^#^*P* < 0.05 and ^##^*P* < 0.005 versus a normal group.

Since Ta-EE ameliorated the symptoms of OA in an OA animal model and exerted *in vivo* anti-inflammatory activity by decreasing the serum levels of inflammatory mediators and proinflammatory cytokines, we further examined the *in vitro* anti-inflammatory activity of Ta-EE using an *in vitro* cell model, LPS-challenged macrophage-like RAW264.7 cells. Given the possibility of false anti-inflammatory activity caused by Ta-EE-mediated cytotoxicity, Ta-EE-mediated cytotoxicity was first examined in RAW264.7 cells. Ta-EE did not exert any cytotoxicity at the examined doses (0–300 *μ*g/ml; Figure 3(a)). The production of inflammatory mediators (nitrite, PGE_2_, and LTB_4_) along with proinflammatory cytokines (IL-1*β* and IL-6) was significantly suppressed by Ta-EE in the LPS-stimulated RAW264.7 cells in a dose-dependent manner (Figures 3(b), 3(c), 3(d), and 3(f)). Moreover, the mRNA expression levels of IL-1*β* and inflammatory genes (iNOS and COX-2) were markedly decreased by Ta-EE in the LPS-stimulated RAW264.7 cells in a dose-dependent manner (Figures 3(g), 3(h), and 3(i)). These *in vitro* results suggest that Ta-EE exerts anti-inflammatory activity by suppressing both the production and mRNA expressions of inflammatory mediators and proinflammatory cytokines in the inflammatory macrophages.

**Figure 4:**
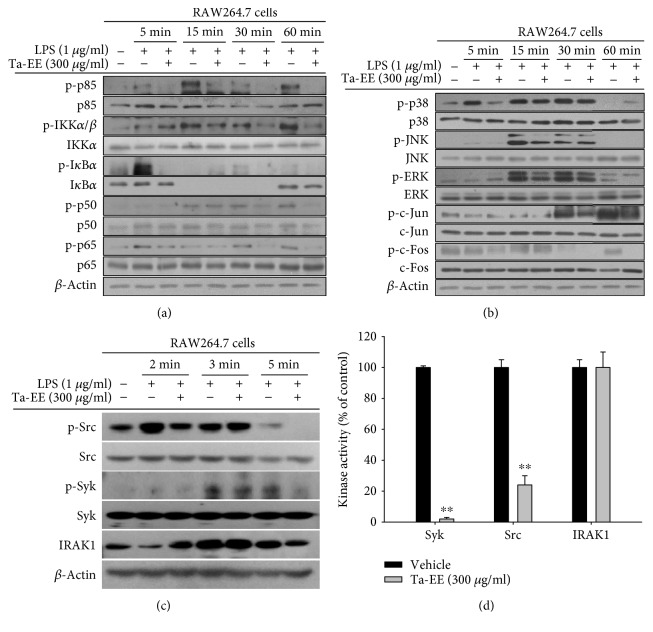
Ta-EE suppressed the activation of the NF-*κ*B and AP-1 signaling pathways in LPS-stimulated RAW264.7 cells. RAW264.7 cells pretreated with Ta-EE (300 *μ*g/ml) for 30 min were treated with LPS (1 *μ*g/ml) for the indicated time, and the total and phosphorylated protein levels of (a) p85, IKK*α*/*β*, I*κ*B*α*, p50, and p65; (b) p38, JNK, ERK, c-Jun, and c-Fos; and (c) Src, Syk, and IRAK1 in the total cell lysates were determined by Western blot analysis. *β*-Actin was used as an internal control. (d) Effects of Ta-EE on the kinase activities of Src, Syk, and IRAK1 were determined by *in vitro* kinase assay using purified Src, Syk, and IRAK1 as described in Materials and Methods. ^∗^*P* < 0.05 and ^∗∗^*P* < 0.005 versus a control group.

Next, we investigated the molecular mechanism by which Ta-EE suppressed inflammatory responses in macrophages. The effects of Ta-EE on the two main inflammatory signaling pathways, the NF-*κ*B and AP-1 signaling pathways, were examined in LPS-stimulated RAW264.7 cells using Western blot analysis. Ta-EE significantly suppressed the activation of the following intracellular signaling molecules in the NF-*κ*B signaling pathway by inhibiting their phosphorylation levels (Figure 4(a)): p85 (15–60 min during LPS treatment), IKK*α*/*β* (15–60 min), I*κ*B*α* (5 min), p50 (30–60 min), and p65 (30–60 min). Ta-EE also suppressed the activation of intracellular signaling molecules in the AP-1 signaling pathway: p38 (5 min), JNK (15 min), ERK (15–60 min), c-Jun (30 min), and c-Fos (60 min). However, the suppressive effect of Ta-EE on the AP-1 signaling pathway was much weaker than on the NF-*κ*B signaling pathway (Figure 4(b)). Therefore, the effects of Ta-EE on the activation of upstream signaling molecules in the NF-*κ*B signaling pathway (Src, Syk, and IRAK1) were further examined in LPS-stimulated RAW264.7 cells. As expected, Ta-EE suppressed the activation of Src, Syk, and IRAK1 by inhibiting the phosphorylation of Src (2 min) and Syk (3–5 min) and the degradation of IRAK1 (2 min; Figure 4(c)). To identify the direct molecular targets of Ta-EE during its anti-inflammatory activity, the kinase activities of both Src and Syk (the phosphorylation of which was both suppressed by Ta-EE) were determined by an *in vitro* kinase assay. Interestingly, Ta-EE significantly suppressed the kinase activities of both Src and Syk (Figure 4(d)), while it did not directly suppress the kinase activity of IRAK1. This indicates that the direct molecular targets of Ta-EE are Src and Syk in the NF-*κ*B signaling pathway. The above findings suggest that Ta-EE exerts anti-inflammatory activity by suppressing the NF-*κ*B signaling pathway via the direct targeting of upstream kinases (Src and Syk) during inflammatory responses in macrophages.

**Figure 5:**
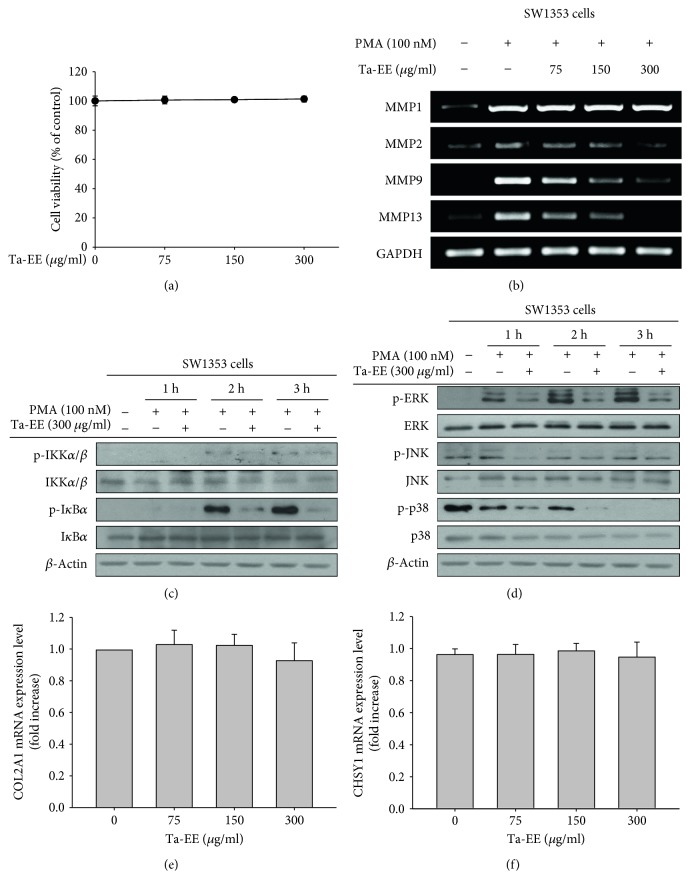
Ta-EE exerted a chondroprotective effect by downregulating MMP gene expression rather than upregulating the gene expressions of COL2A1 and CHSY1 in SW1353 cells. (a) SW1353 cells were treated with Ta-EE (0–300 *μ*g/ml) for 24 h, and cell viability was measured by MTT assay. (b) SW1353 cells pretreated with Ta-EE (0–300 *μ*g/ml) for 30 min were treated with PMA (100 nM) for 6 h, and the mRNA expression levels of MMP1, MMP2, MMP9, and MMP13 in the cells were measured by semiquantitative PCR. SW1353 cells pretreated with Ta-EE (300 *μ*g/ml) for 30 min were treated with PMA (100 nM) for the indicated time, and the total and phosphorylated protein levels of (c) IKK*α*/*β* and I*κ*B*α* and (d) ERK, JNK, and p38 in the total cell lysates were determined by Western blotting. *β*-Actin was used as an internal control. (e) SW1353 cells were treated with Ta-EE (0–300 *μ*g/ml) for 6 h, and the mRNA expression levels of COL2A1 and CHSY1 in the cells were measured by quantitative real-time PCR.

The primary cells and tissues damaged during OA pathogenesis are chondrocytes and articular cartilage because chondrocytes are located in the superficial areas of articular cartilage. Therefore, we investigated the effect of Ta-EE on chondrocytes using chondrosarcoma cells (SW1353 cell line). Ta-EE did not exhibit any cytotoxicity towards SW1535 cells at doses ranging from 0 to 300 *μ*g/ml (Figure 5(a)). Extracellular matrix metalloproteinases (MMPs), zinc-dependent endopeptidases, have multiple biological roles in macromolecular protein turnover and tissue remodeling [55]. Most MMPs are produced in the chondrocytes and are responsible for the degradation of various pericellular and interterritorial proteins in the extracellular matrix of articular cartilage. The expressions of MMP genes have been reported to be upregulated in OA sera and synovial fluids, leading to the degradation and remodeling of proteins in the cartilage extracellular matrix [56, 57]. Therefore, the effects of Ta-EE on the expressions of MMP genes were examined in SW1353 cells by semi-quantitative PCR. We selected MMP1, MMP2, MMP9, and MMP13 as primary targets because these MMPs are collagenases and gelatinases that degrade collagens and gelatins, which are abundant in the extracellular matrix of articular cartilage. Ta-EE dramatically downregulated the gene expressions of MMP2, MMP9, and MMP13 but not that of MMP1 in PMA-stimulated SW1353 cells in a dose-dependent manner (Figure 5(b)).

We also examined the molecular mechanism by which Ta-EE downregulated the expressions of the above MMP genes in PMA-stimulated SW1353 cells. Similar to those in Figures 4(a) and 4(b), the effects of Ta-EE on the activation of intracellular signaling molecules in the NF-*κ*B and AP-1 signaling pathways were examined by Western blot analysis. Ta-EE markedly suppressed the activation of IKK*α*/*β* (2-3 h) and I*κ*B*α* (2-3 h) in the NF-*κ*B signaling pathway by inhibiting their phosphorylation (Figure 5(c)). Ta-EE also suppressed the activation of ERK (1–3 h), JNK (1–3 h), and p38 (1-2 h) in the AP-1 signaling pathway by inhibiting their phosphorylation (Figure 5(d)). These results indicate that Ta-EE downregulated the expressions of MMP genes by suppressing both the NF-*κ*B and AP-1 signaling pathways in the chondrocytes of PMA-stimulated SW1353 cells.

Next, we investigated the effects of Ta-EE on the expressions of genes known to produce extracellular matrix in articular cartilage. The expressions of collagen type II alpha I (COL2A1) and chondroitin sulfate synthase 1 (CHSY1) were examined by quantitative real-time PCR in Ta-EE-treated SW1353 cells. Unexpectedly, Ta-EE did not affect the gene expression of COL2A1 (Figure 5(e)) or CHSY1 (Figure 5(f)), suggesting that the chondroprotective function of Ta-EE is exerted by preventing cartilage degradation rather than by facilitating cartilage generation.

## 4. Conclusion

**Figure 6:**
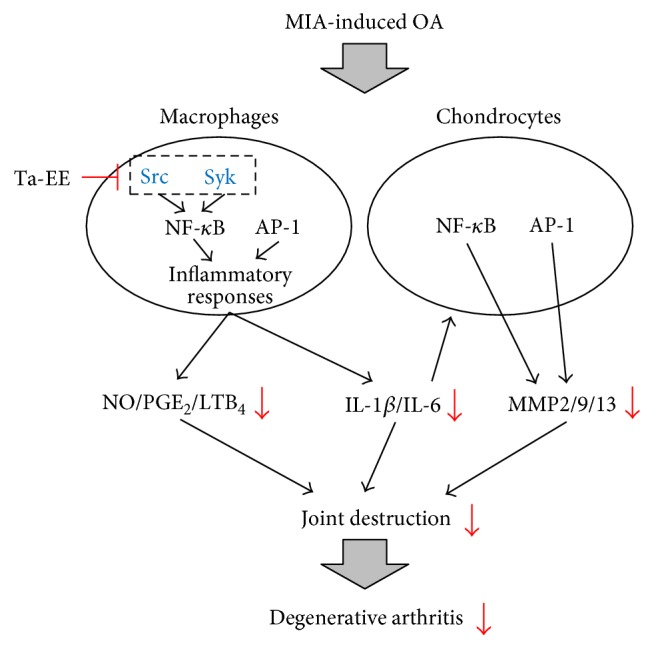
Proposed model showing the inhibitory pathways associated Ta-EE-mediated anti-inflammatory and chondroprotective activities in OA pathogenesis.

In conclusion, we investigated the *in vivo* pharmacological effects of Ta-EE on OA pathogenesis using an experimental OA animal model and the underlying molecular mechanisms of these effects using *in vitro* macrophage and chondrocyte cellular models. Ta-EE effectively ameliorated the symptoms of OA and delayed the onset and progression of OA without any toxicity or side effects in MIA-induced OA rats. These pharmacological effects were attributed to the anti-inflammatory activity of Ta-EE, which was exerted through the inhibition of the two most critical signaling pathways in inflammatory response: the NF-*κ*B and AP-1 signaling pathways in macrophages. Specifically, Ta-EE directly targeted Src and Syk in the NF-*κ*B signaling pathway. In addition, Ta-EE exerted chondroprotective activity by downregulating the gene expressions of various MMPs via the suppression of both the NF-*κ*B and AP-1 signaling pathways rather than by upregulating the expressions of extracellular matrix-generating genes in chondrocytes. The findings of this study are summarized in Figure 6. The results provide evidence to support the correlation between OA pathogenesis and inflammatory response and demonstrate the anti-inflammatory and chondroprotective activities of Ta-EE in OA pathogenesis. Therefore, the findings of this study improve our understanding of the relationship between OA pathogenesis and inflammation and provide new insights to develop drugs to prevent and treat human inflammatory diseases, including OA.

## Figures and Tables

**Table 1:** Nucleic acid sequences of the primers used for PCR.

Target		Sequence (5′ to 3′)
Semiquantitative real-time PCR		
MMP1	Forward	CCCAGCGACTCTAGAAACACA
	Reverse	CTGCTTGACCCTCAGAGACC
MMP2	Forward	ACGACCGCGACAAGAACTAT
	Reverse	CTGCAAAGAACACAGCCTTCTC
MMP9	Forward	CAGTACCGAGAGAAAGCCTA
	Reverse	ACTGCAGGATGTCATAGGTC
MMP13	Forward	GAAATGCAGTCTTTCTTCGG
	Reverse	GCCTTTTCGACTTCAGAATG
GAPDH	Forward	CACTCACGGCAAATTCAACGGCAC
	Reverse	GACTCCACGACATACTCAGCAC

Quantitative real-time PCR		
IL-1*β*	Forward	TAGAGCTGCTGGCCTTGTTA
	Reverse	ACCTGTAAAGGCTTCTCGGA
iNOS	Forward	GGAGCCTTTAGACCTCAACAGA
	Reverse	TGAACGAGGAGGGTGGTG
COX-2	Forward	CACTACATCCTGACCCACTT
	Reverse	ATGCTCCTGCTTGAGTATGT
COL2A1	Forward	GCAACGTGGTGAGAGAGGAT
	Reverse	CCTGTCGTCCGGGTTCAC
CHSY1	Forward	ATTGTCATGCAGGTCATGGA
	Reverse	CTCACAGGGACCGTCATTTT
GAPDH	Forward	CAATGAATACGGCTACAGCAAC
	Reverse	AGGGAGATGCTCAGTGTTGG

## Abbreviations

OA: Osteoarthritis
Ta-EE: *Tabebuia avellanedae* ethanol extract
MIA: Monoiodoacetate
NO: Nitric oxide
ROS: Reactive oxygen species
H_2_O_2:_: Hydrogen peroxide
NF-*κ*B: Nuclear factor-kappa B
AP-1: Activator protein-1
IL: Interleukin
iNOS: Inducible nitric oxide synthase
COX-2: Cyclooxygenase-2
PGE_2:_: Prostaglandin E_2_
PMA: Phorbol 12-myristate 13-acetate
Pc-LE: *Perna canaliculus* lipid extract
MTT: 3-(4,5-Dimethylthiazol-2-yl)-2,5-diphenyltetrazolium bromide
SDS: Sodium dodecyl sulfate
ELISA: Enzyme-linked immunosorbent assay
PCR: Polymerase chain reaction
GAG: Glycosaminoglycan
IKK*α*/*β:*: I*κ*B kinase alpha/beta
I*κ*B*α:*: NF-*κ*B *inhibitor alpha*
JNK: c-Jun N-terminal kinase
ERK: Extracellular signal-regulated kinase
IRAK: Interleukin-1 receptor-associated *kinase*
MMP: Matrix metalloproteinase
COL2A1: Collagen type II alpha 1
CHSY1: Chondroitin sulfate synthase 1.
